# Characteristic cortico-cortical connection profile of human precuneus revealed by probabilistic tractography

**DOI:** 10.1038/s41598-023-29251-2

**Published:** 2023-02-02

**Authors:** Tatsuya Jitsuishi, Atsushi Yamaguchi

**Affiliations:** Department of Functional Anatomy, Graduate School of Medicine, Chiba University, 1-8-1 Inohana, Chuo-ku, Chiba, 260-8670 Japan

**Keywords:** Computational biology and bioinformatics, Neuroscience, Physiology, Psychology, Anatomy, Neurology

## Abstract

It is generally hypothesized that functional connectivity (FC) reflects the underlying structural connectivity (SC). The precuneus is associated with highly integrated cognitive functions. However, our understanding of the structural connections that could underlie them is limited. This study aimed to characterize the cortico-cortical connections by probabilistic tractography. The precuneus corresponds to the five cortical areas (7Am, PCV, 7Pm, 7m, POS2) on the HCP MMP atlas. We first conducted the atlas-based probabilistic tractography. The anterior part (7Am) was strongly connected to the sensorimotor region. The dorsal part (7Am, 7Pm) was highly connected with the adjacent parietal and temporal cortex, while the ventral part (PCV, 7m) showed strong connections with the adjacent posterior cingulate and medial prefrontal cortex. The most posterior part (POS2) was explicitly connected to the visual cortex. In addition, there was a correlation between SC and resting-state fMRI connectivity (Spearman’s rank correlation coefficient = 0.322 ± 0.019, *p* < 0.05 corrected at subject level). Collectively, the current study revealed the characteristic connectional profile of precuneus, which could shed light on the structural heterogeneity for the future functional analyses.

## Introduction

The precuneus is an association area on the posteromedial cortex that borders the somatosensory, the posterior parietal, the posterior cingulate, and the visual cortex. It is involved in highly integrated cognitive functions, including visuospatial processing, episodic memory retrieval, self-relevant processing, and consciousness^1,2^. Generally, it is believed the functional connectivity (FC) patterns might reflect the underlying structural connectivity (SC)^3–5^. Yet, our understanding is limited on the anatomical connections of precuneus that could underlie the cognitive functions. This study aimed to characterize the cortico-cortical connections by probabilistic tractography and then to analyze the SC-resting state FC (RSFC) relationship.

Based on the traditional anatomical descriptions, the precuneus is bordered by the marginal branch of the cingulate sulcus anteriorly, by the parieto-occipital fissure posteriorly, and by the sub-parietal sulcus inferiorly^1,6,7^. The territory of precuneus is compatible with the mesial extent of Brodmann area 7 (BA7)^1,8–10^. Several human brain atlases have been developed, which parcellate human cerebral cortices into homologous areas by aerial quantitative measures, including cortical thickness and volume, microstructural diffusivity, blood perfusion, and functional activity. The publicly available brain atlases are advantageous in allowing for direct comparisons across studies^11^. To perform atlas-based tractography, we then used the multi-modal parcellation of the Human Connectome Project (HCP MMP atlas), an updated map of the human cerebral cortex. The HCP MMP atlas consists of 180 distinct areas per hemisphere delineated by a combination of multi-modal neuroimaging data, including cortical thickness, myelination, resting-state, and task-based fMRI^12^. However, the precise tractograhy-based structural connectome using HCP diffusion MRI data has not been released until now. We here confined the territory of precuneus to the mesial extent of BA7^1,13^, which is compatible with five cortical areas (7Am, PCA, 7Pm, 7m, POS2) of the HCP MMP atlas^14,15^.

Several studies have reported the SC and FC of human precuneus by diffusion MRI (dMRI) and functional MRI (fMRI) analyses, respectively. Margulies et al.^16^ showed the human precuneus consists of three distinct sub-divisions by resting-state fMRI (rs-fMRI), similar to those of macaque monkeys: anterior, central, and posterior precuneus. By a K-means clustering algorithm of RSFC, Zhang et al.^17^ showed the precuneus is functionally divided into the dorsal-anterior, dorsal-posterior, and ventral subregions, involved in spatially guided behaviors, mental imagery, episodic memory with self-related processing, and the default mode network, respectively. Wang et al.^18^ identified three subregions in the human precuneus, two in the dorsal and one subregion in the ventral precuneus, by analyzing both anatomical connectivity and task-dependent coactivation. Luo et al.^19^ demonstrated the precuneus is functionally subdivided into six symmetrical and connected parcels by the developed eigen clustering (EIC) approach with fMRI data.

dMRI quantifies the restriction of isotropic diffusive water movement to infer SC by fiber tracking, while fMRI quantifies the blood oxygen level-dependent (BOLD) signals to infer FC through neurovascular coupling^20–22^. The structural connectivity strength was estimated through the counts of streamline endpoints per voxel produced by tractography, while the functional connectivity strength was measured by z-value to represent neural correlates between two points. The algorithms of fiber tracking by dMRI are broadly classified into deterministic and probabilistic tractography. Generally, the probabilistic tractography is considered preferable for reconstructing and dissecting individual white matter tracts to quantify the probability of each reconstructed pathway^23^. To obtain all possible connections between cortical regions, we employed the probabilistic method with Mrtrix3 (https://www.mrtrix.org)^24^. Mrtrix3 algorithm reportedly outperforms DTI (diffusion tensor imaging) and other alternatives methods of fiber tracking in crossing fiber regions^25,26^.

In the present study, we first examined the atlas-based cortico-cortical connections of human precuneus by probabilistic tractography with the HCP MMP atlas. Then we analyzed the relationship between SC and RSFC of the precuneus.

## Material and methods

### Data source and MRI acquisition

The neuroimaging data of healthy young adults (WU-Minn HCP open Data, HCP S1200 release), including structural MRI (T1-weighted image), diffusion MRI (dMRI), and resting-stage fMRI (rs-fMRI), were publicly obtained from the Human Connectome Project (HCP) database ConnectomeDB (https://www.humanconnectome.org)^27^. The 33 healthy young adult unrelated subjects’ data were analyzed in the present study from the HCP database.

The HCP consortium has developed a common MRI acquisition protocol in the HCP reference manual (https://humanconnectome.org/storage/app/media/documentation/s1200/HCP_S1200_Release_Reference_Manual.pdf). Briefly, Siemens Skyra 3T scanner was used to acquire multi-modal MRI data^28^. The structural data (T1-weighted image) consisted of one 0.7 mm isotropic scan in each subject. For dMRI, data was acquired with a spatial resolution (1.25 mm isotropic), multiple angular contrasts (b = 1000, b = 2000, and b = 3000 s/mm^2^), and high SNR (multiple averages and dense sampling in q-space, giving 570 volumes). For rs-fMRI, total of 4 fMRI runs were acquired in 2 mm isotropic resolution with a TR of 0.72 s for each 15 min period. Then, HCP MRI data were preprocessed by a common automated preprocessing framework^29^.

### HCP MMP atlas and 22 Cortical_Divisions

The HCP MMP (Human connectome project multi-modal parcellation) atlas consists of 180 distinct areas per hemisphere from hundreds of HCP subjects, which is delineated by a combination of multi-modal neuroimaging data, including cortical thickness, myelination, resting-state, and task-based fMRI^12^. The 180 cortical areas were grouped into 22 Cortical_Divisions by several criteria of geographic proximity and functional similarities for organizational purposes. Each Cortical_Division includes a set of geographically contiguous cortical areas, sharing common properties based on architecture, task-fMRI profiles, and/or functional connectivity. The table of the 22 Cortical_Divisions with corresponding 180 area is shown in Supplementary Fig. S2.

### Probabilistic tractography

To conduct probabilistic tractography with each subject’s dMRI data, we utilized the MRtrix3 software package (http://www.mrtrix.org) based on the protocol for HCP datasets (https://mrtrix.readthedocs.io/en/0.3.16/tutorials/hcp_connectome.html)^24^. We first generated a tissue-segmented image appropriate for ACT (Anatomically-Constrained Tractography)^30^. Next, we estimated the response functions from the preprocessed diffusion-weighted images to estimate fiber orientation distributions (FOD) based on the constrained spherical deconvolution (CSD) using iFOD2 algorithm. We then conducted tractography using “tckgen” command (seed = whole brain, default step size, maximum harmonics order = 8, 100 million streamlines with maximum tract length = 250 mm and minimal tract length = 10 mm, termination criteria: exit the brain or when the CSD fiber-orientation distribution amplitude was 0.06) with Spherical-deconvolution Informed Filtering of Tractograms 2 (SHIFT2) to improve the quantitative nature of whole-brain streamlines reconstructions^31^. The HCP-MMP atlas (https://figshare.com/articles/HCP-MMP1_0_projected_on_fsaverage/3498446) was used for the parcellation of the cerebral cortex^29^.

### Streamline connection matrix

The detailed pipeline for the connection matrix of streamline count is shown in Supplementary Fig. S1. Briefly, after conducting a whole-brain tractography at the subject level, we created the group-average structural connection matrix based on the protocol of the BATMAN_tutorial (Basic and Advanced Tractography with MRtrix for All Neurophiles, https://osf.io/pm9ba). The HCP MMP atlas annotations were labeled onto each subject’s brain image in FreeSurfer^32^ with the publicly available HCP MMP atlas (https://figshare.com/articles/HCP-MMP1_0_projected_on_fsaverage/3498446). Then to address the connection profile of streamlines seeded from 5 precuneus ROIs (7Am, PCV, 7Pm, 7m, POS2), we obtained the connection matrix to represent the streamline count between each cortical area (end-to-end) in the HCP MMP atlas at the subject level [5 Precuneus ROIs × Whole-brain areas; 5 × 180 × 2 (left and right hemisphere)]. The group-average end-to-end connection matrix indicated the average connection matrix across 33 subjects.

### Density map of streamline endpoint

The detailed pipeline for the density map of the streamline endpoint was shown in Supplementary Fig. S1. Briefly, the group-average template brain across 33 subjects was produced by FreeSurfer (command; mriaverage). After the tractogram was normalized to MNI standard space at the subject level, the maps of streamline endpoint (Mrtrix3 command; tckmap) seeded from 5 precuneus ROIs to the whole brain were obtained at the group level [5 Precuneus ROIs × Whole brain; 5 × 2 (right and left hemisphere) = 10 maps]. Then we produced the density map by rendering streamline endpoints onto the group-average template brain by Workbench (https://www.humanconnectome.org/software/connectome-workbench). This endpoint density map represents the streamline endpoints to each cortical area from 5 precuneus ROIs per hemisphere on the group-average template brain, respectively.

### Resting-state functional MRI (rs-fMRI)

The rs-fMRI data were downloaded from the rs-fMRI FIX-Denoised (Extended) Package of the HCP S1200 release. This package includes preprocessed data that had been registered and denoised with the FIX ICA-based automated method^33^. Functional connectivity (FC) was measured with the CONN-fMRI toolbox in the MATLAB-based cross-platform Statistical Parametric Mapping (SPM12) (https://web.conn-toolbox.org/).

In brief, rs-fMRI data were processed by CONN through slice-timing correction, realignment, normalization, and spatial smoothing. The seed-based connectivity (SBC) analysis of fMRI basically computes the temporal correlation of blood oxygen level-dependent (BOLD) signals between a distinct seed region and other areas using a General Linear Model (GLM) approach with hemodynamic response function (HRF). For the parcellation of cortical areas, the publicly available HCP MMP1.0 atlas (https://figshare.com/articles/HCP-MMP1_0_projected_on_fsaverage/3498446) was incorporated to CONN for FC analyses. At subject level, all FC measures were obtained from CONN as first-level analyses. Subject-specific contrast images that reflect standardized correlation coefficient (*r*) were processed, which were converted to the normally distributed variable by Fisher's *z*-transformation for further analyses. As second-level analyses, the one-sample *t*-tests of the correlation coefficients were calculated to obtain positive and negative FC maps within groups. Group-level differences in FC were computed by an Analysis of Variance (ANOVA). After automatically generated for ANOVAs in the SPM software, a set of standard *t*-contrasts was utilized to assess the main effect of the group and the interaction between groups. The threshold for all analyses was set as a voxel-wise *p* < 0.001 uncorrected and a cluster-level *p* < 0.05 FDR corrected (https://web.conn-toolbox.org/). The regions with significant neural correlates were rendered on the template brains in the CONN platform.

### Laterality Index (LI)

The LI value is computed using the following formula for each pairwise connectivity of SC and RSFC, respectively^34^. LI = [Intra-hemispheric value−Inter-hemispheric value]/[|Intra-hemispheric value| +|Inter-hemispheric value|], in which LI varies from − 1 to 1.

### Statistical analysis

To assess the SC-RSFC correlation, SC values were resampled to a normal distribution by *z*-transformation. Furthermore, negative FC values were set to 0 following previous works^35,36^. SC-RSFC correlation (Fig. 5A) was estimated with Spearman's rank correlation coefficient at the whole brain level (correlation of the whole brain) and at each precuneus ROI level (correlation between each precuneus ROI and whole-hemisphere)^37^. The *p* < 0.001 at subject level (uncorrected) and *p* < 0.05 at group level (FDR corrected) was considered statistically significant. The intra-precuneus connections (seed and target region within the precuneus) were excluded for estimating the SC-RSFC correlation. Data were analyzed using the EZR (R package statistical program) ^38^.

### Ethical statement

All MRI imaging data in the present study was publicly obtained from the Human Connectome Project (https://www.humanconnectome.org). All methods and protocols, approved by the Research Ethics Committee of Chiba University School of Medicine, were performed in accordance with relevant guidelines and regulations.

## Results

### Neuroanatomy and parcellation by HCP MMP atlas

**Figure 1:**
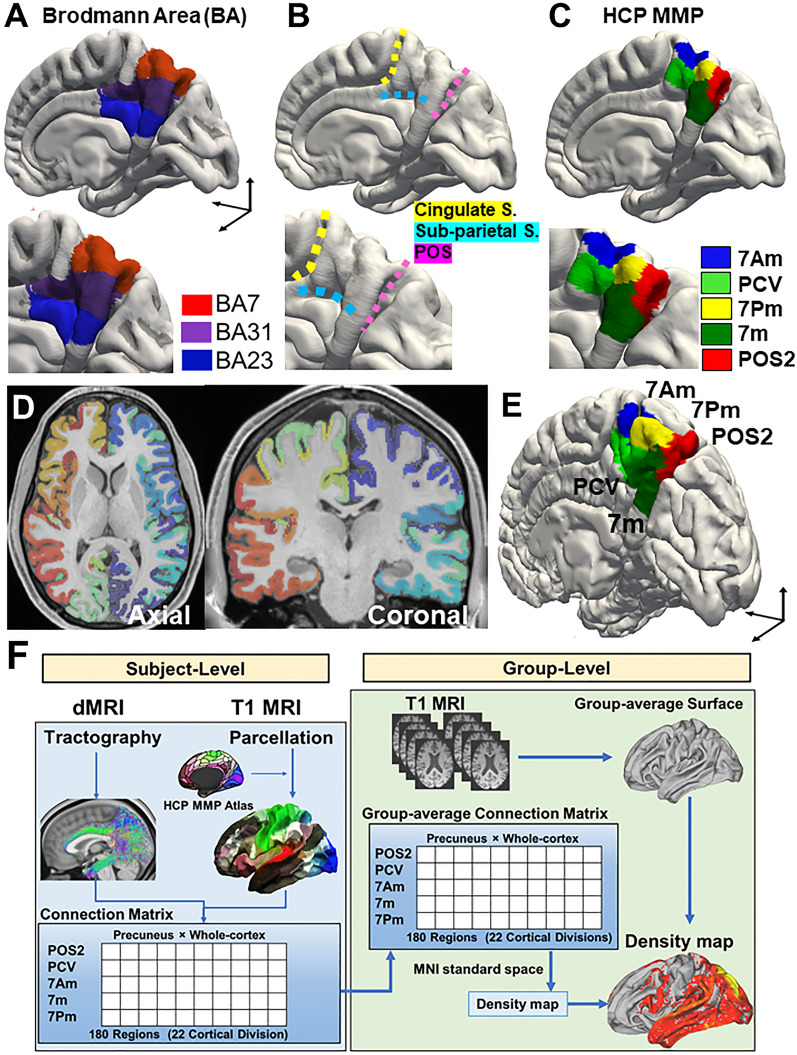
Neuroanatomy and parcellation of precuneus. (**A**) The territory of Brodmann areas 7, 23, and 31 (in the FreeSurfer atlas) were rendered on the template brain (Pial surface). (**B**) The precuneus is bordered by the marginal branch of the cingulate sulcus (Cingulate S.) anteriorly, by the sub-parietal sulcus (Sub-parietal S.) inferiorly, and by the parieto-occipital sulcus (POS) posteriorly. (**C**) Parcellation of precuneus by HCP MMP atlas in the template brain. (**D**) Representative parcellation of single subject’s brain images (axial and coronal) by HCP MMP atlas (Subject ID: HCP #102614). (**E**) Representative parcellation of precuneus by HCP MMP atlas in a single subject’s brain image (Subject ID: HCP #103414). (**F**) Flow chart showing the workflow to produce density map of streamline endpoint by probabilistic tractography. At the subject-level, diffusion MRI (dMRI) data were analyzed to produce a structural connection matrix by probabilistic tractography. T1 MRI data were analyzed to annotate labels in the HCP-MMP atlas and to produce a group average white surface brain. After producing a group-level structural connection matrix based on the HCP MMP atlas, the endpoint density map was rendered on the group-average white matter surface in MNI standard space. Details of this process are shown in Supplementary Fig. S1.

The precuneus, corresponding to the mesial extent of BA7, is bordered by the three sulci, including the marginal branch of the cingulate sulcus (Cingulate S.) anteriorly, the sub-parietal sulcus (Sub-parietal S.) inferiorly, and the parieto-occipital sulcus (POS) posteriorly (Fig. 1A, B)^6,8^. This territory is compatible with the five cortical areas of the HCP MMP atlas, including anterior-dorsal (7Am), anterior-ventral (PCV), posterior-dorsal (7Pm), posterior-ventral area (7m), and the anterior wall of the parieto-occipital sulcus (POS2) (Fig. 1C).

In Fig. 1D and and E, we showed the representative parcellation of the cerebral cortex and precuneus by HCP MMP atlas in a single subject’s brain images, respectively. The flow chart in Fig. 1F indicates the simplified protocol to conduct the probabilistic tractography with the parcellation of HCP MMP atlas, which was used to make the connection matrix and the endpoint density map of streamline at the group level. The more detailed pipeline is shown in Suppl. Fig. S1.

### Intra-hemispheric SC of precuneus

**Figure 2:**
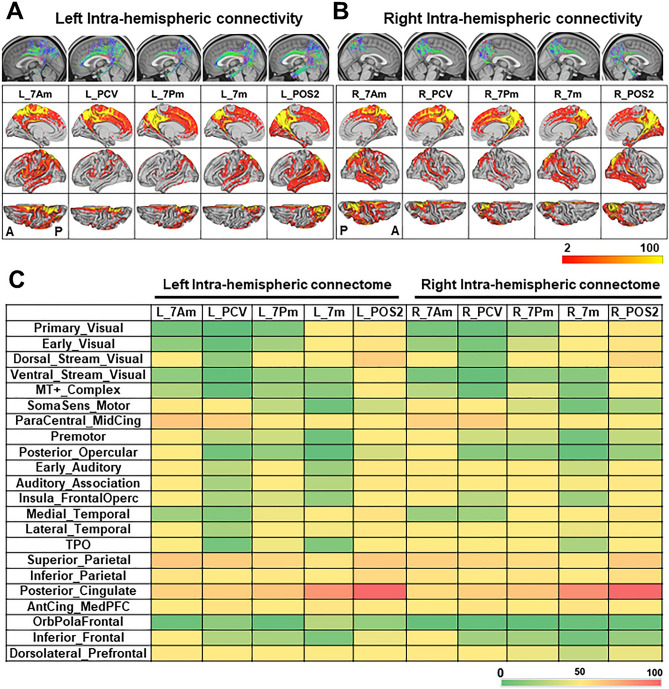
Intra-hemispheric structural connectivity (SC) of precuneus. (**A**, **B**) Tractogram and intra-hemispheric density map of streamline endpoint, seeded from each precuneus ROI by probabilistic tractography. Top panels are tractograms seeded from each precuneus ROI in the left (**A**) and right hemisphere (**B**), respectively. Brain regions with the density map of streamline endpoint were projected onto the group-average template brain (white matter surface) in the left (**A**) and right hemisphere (**B**), respectively. Medial, lateral, and top views of the hemisphere was shown in each lane. The color scale bar represents the group-average endpoint count/voxel (range; 2–100). A, anterior; P, posterior. (**C**) Color map showing the intra-hemispheric group-average connection matrix of streamline count to connect each precuneus ROI (7Am, PCV, 7Pm, 7m, POS2) and the 22 Cortical Divisions of the HCP MMP atlas. The rows show the 22 Cortical Divisions of HCP MMP atlas, while the lines show the 5 precuneus ROIs in the left (L) and right (R) hemisphere, respectively. The color scale bar indicates the ratio (%) of maximum streamline count/area (i.e., R_Posterior_Cingulate - R_POS2). The details of 22 Cortical Divisions in HCP MMP atlas are shown in Supplementary Fig. S2 with the abbreviations.

We then investigated the intra-hemispheric SC of precuneus by probabilistic tractography, using diffusion MRI data in the HCP. The group-average density maps of the streamline endpoint, seeded from the 5 precuneus ROIs (7Am, PCV, 7Pm, 7m, POS2), were rendered on the group-average brain surface in the left- and right-hemisphere, respectively (Fig. 2A, B). The color map in Fig. 2C shows the group-average connection matrix of the streamline count to connect 5 precuneus ROIs with 22 Cortical Divisions of the HCP MMP atlas in the left and the right hemisphere, respectively. The details of the 22 Cortical Divisions are shown in Suppl. Fig. S2^29,39^.

The anterior-dorsal area (7Am) was connected extensively to the frontal, parietal, lateral temporal lobe, and adjacent cingulate cortices, including ParaCentral_MidCing, Superior_Parietal, Inferior_Parietal, Posterior_Cingulate, and Dorsolateral_Prefrontal cortical division (Fig. 2A–C). The anterior-ventral area (PCV) was connected to the frontal, parietal lobe, and adjacent cingulate cortex, including ParaCentral_MidCing, Superior_Parietal, Inferior_Parietal, and Posterior_Cingulate division. In addition, the right PCV has more connections laterally to the parietal and temporal lobe (auditory areas). The posterior-dorsal area (7Pm) was connected broadly to the parietal, temporal lobe, and adjacent cingulate cortices, including Superior Parietal, Inferior Parietal, Posterior_Cingulate, and Dorsal_stream_visual division. The posterior-ventral area (7m) was connected to the parietal, temporal lobe, and adjacent cingulate cortices, including Posterior_Cingulate, Superior_Parietal, Medial_Temporal, and AntCing_MedPFC division. The anterior wall of POS (POS2), the most posterior part, was connected extensively to the frontal, parietal, temporal lobe, and adjacent cingulate cortices, including Posterior_Cingulate, Dorsal_Stream_Visual, Superior_Parietal, Inferior_Parietal, and Medial_Temporal division. In particular, POS2 was specifically linked to the adjacent visual regions, including primary, early, and high-level visual areas.

### Intra-hemispheric resting-state FC (RSFC) of precuneus

**Figure 3:**
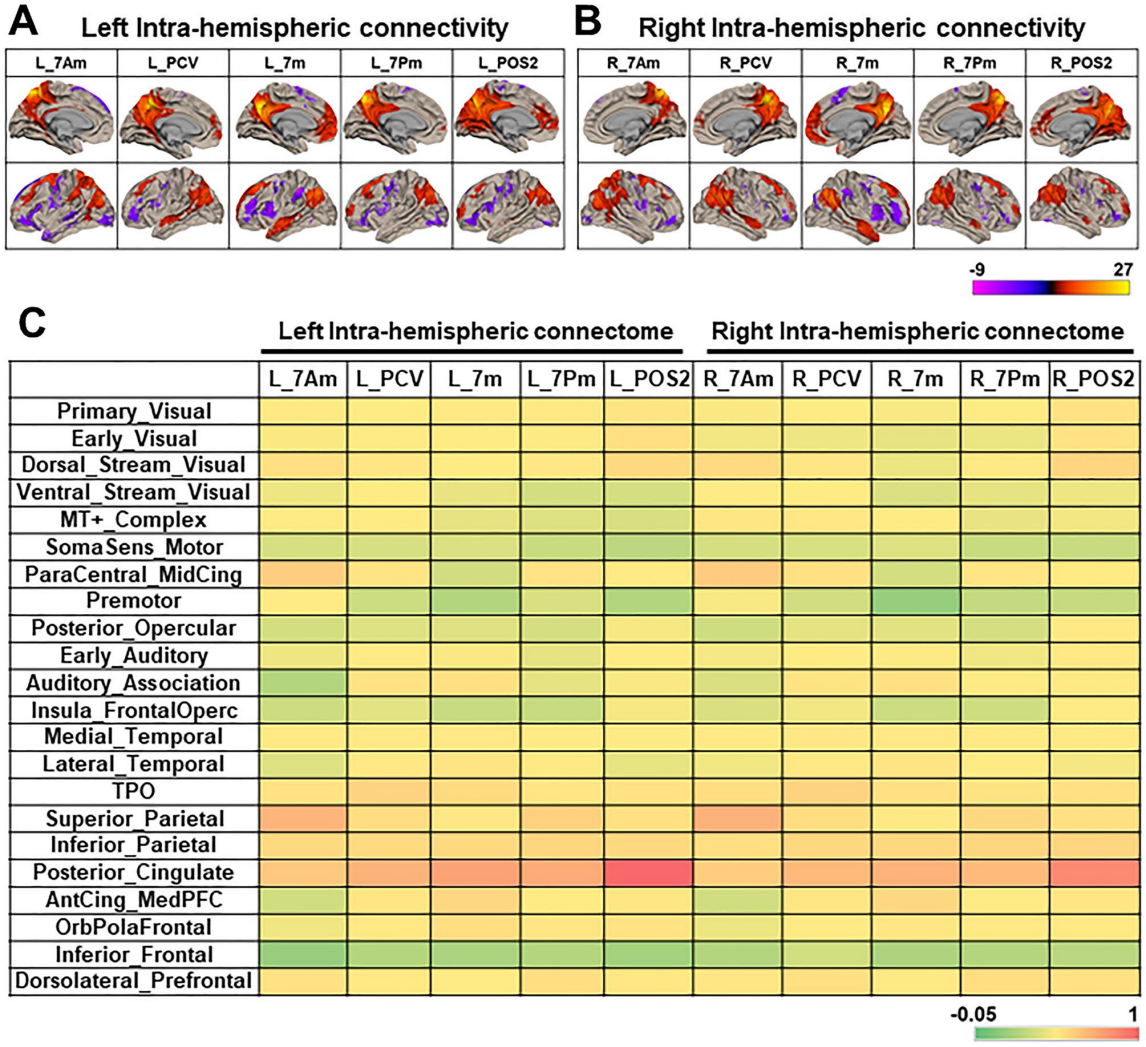
Intra-hemispheric functional connectivity (FC) of precuneus. (**A**, **B**) Seed-based RSFC of each precuneus ROI (7Am, PCV, 7Pm, 7m, POS2) in the left (**A**) and right (**B**) hemisphere, respectively. The regions with significant neural correlate at second-level analysis (voxel-level *p* < 0.001 uncorrected, cluster-level *p* < 0.05 FDR corrected) were rendered on the template brain (white matter surface). The color bar represents T-values, in which warmer colors indicate higher T-values. (**C**) Color map showing the intra-hemispheric group-average connection matrix of FC strength (group-average pairwise ROI correlation) between each precuneus ROI (7Am, PCV, 7Pm, 7m, POS2) and the 22 Cortical Divisions of the HCP MMP atlas. The color scale bar indicates the group-average correlation coefficient (− 0.05 < r < 1). The rows show the 22 Cortical Divisions of the HCP MMP atlas, while the lines show the 5 seed regions of the precuneus in the left (L) and right (R) hemisphere, respectively. The details of 22 Cortical_Divisions in HCP MMP atlas are shown in Supplementary Fig. S2.

We then analyzed the seed-based RSFC with rs-fMRI data of the same subjects used in the probabilistic tractography. The regions with significant neural correlates (voxel-level *p* < 0.001 uncorrected, *p* < 0.05 cluster-level corrected) were rendered on the template brain, showing the characteristic neural correlates of each seed of precuneus (Fig. 3A, B). The heat map in Fig. 3C shows the group-average functional connection matrix between each seed of precuneus and 22 Cortical Divisions of HCP MMP atlas.

The anterior-dorsal area (7Am) shows higher RSFC strength with the prefrontal cortex, superior and inferior parietal lobe, and adjacent posterior cingulate cortex, including Superior_Parietal, Inferior_Parietal, Posterior_Cingulate, and ParaCentral_MidCing division. The anterior-ventral area (PCV) has higher RSFC strength with the prefrontal cortex, inferior parietal lobe, temporal lobe, and adjacent posterior cingulate cortices, including Posterior_Cingulate, TPO, and Inferior_Parietal division. The posterior-dorsal area (7Pm) has higher RSFC strength with the prefrontal cortex and inferior parietal lobe, including Posterior_Cingulate, Superior_Parietal, and Inferior_Parietal cortical division. The posterior-ventral area (7m) has higher RSFC strength with the prefrontal cortex, superior and inferior parietal lobe, and adjacent posterior cingulate cortex, including Posterior_Cingulate, Inferior_Parietal, Auditory_Association, AntCing_MedPFC, and OrbPolaFrontal division. The POS2 has higher RSFC strength with the prefrontal cortex, parietal lobe, and adjacent posterior cingulate cortex, including Posterior_Cingulate, Primary_visual, Dorsol_Stream_Visual, Ventral_Stream_Visual, Superior_Parietal, and Inferior_Parietal division. In particular, the POS2 was specifically related with adjacent visual areas.

**Figure 4:**
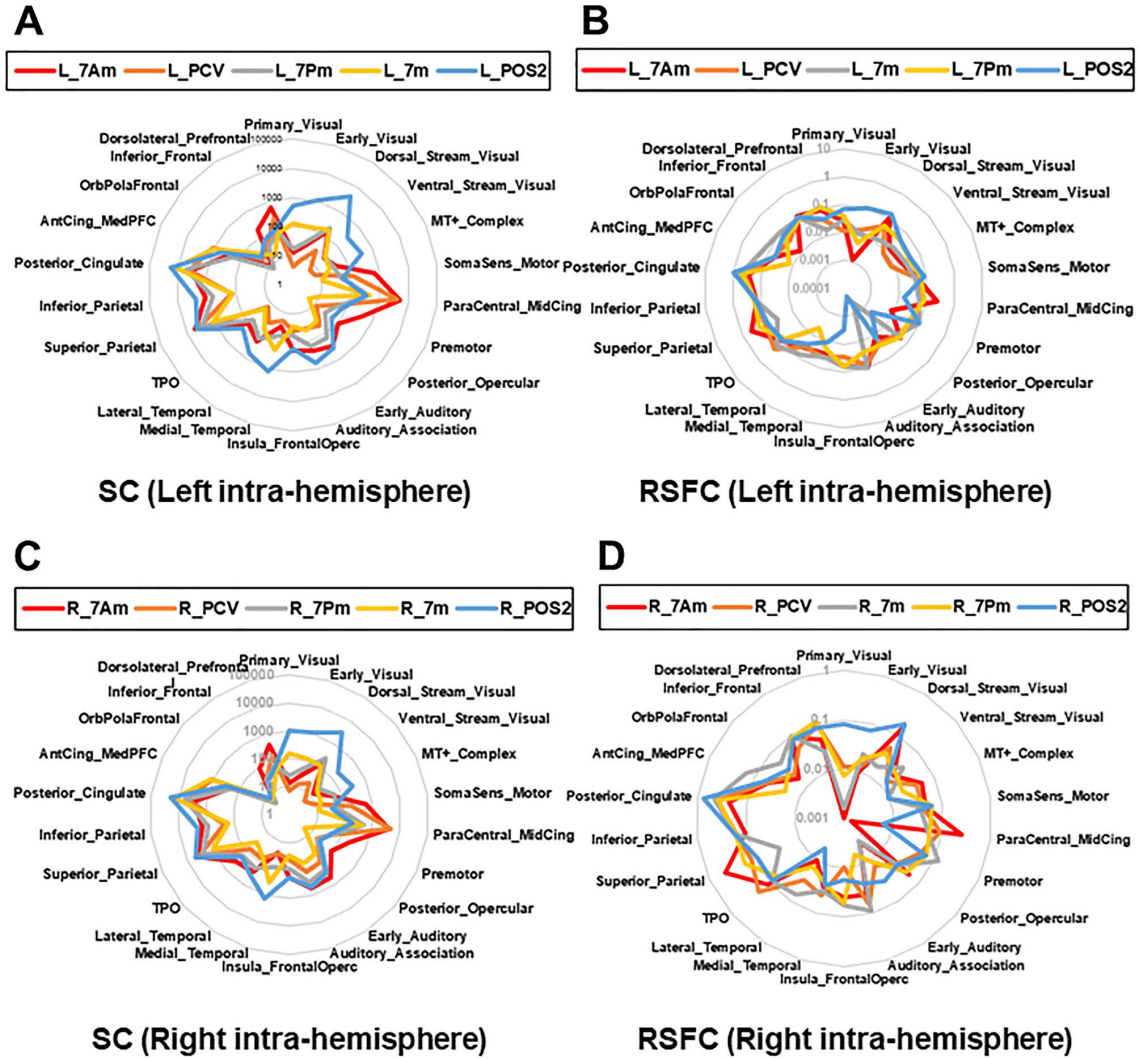
Radar plots showing the connectional profile of SC and RSFC in the left and right hemisphere. (**A**, **B**) Radar plot showing the intra-hemispherical group-average connectional profile of the SC (**A**) and the RSFC (**B**) between each precuneus ROI (7Am, PCV, 7Pm, 7m, POS2) and the 22 Cortical Divisions in the left hemisphere, respectively. (**C**, **D**) Radar plots showing the intra-hemispherical group-average connectional profile of the SC (**C**) and the RSFC (**D**) in the right hemisphere, respectively. Scale; SC (streamline count/area), RSFC (Fisher's z transformed pairwise correlation converted to Log scale). The details of 22 Cortical_Divisions in HCP MMP atlas are shown in Supplementary Fig. S2.

We then showed the radar graphs to indicate the characteristic SC and RSFC in the left (Fig. 4A, B) and right hemisphere (Fig. 4C, D), respectively. In particular, the anterior part (7Am, PCV) has higher connectivity to the frontal cortex (Dorsolatelal_Prefrontal, ParaCentral_MidCing) and Superior_Parietal, while POS2 has connections broadly with strong connectivity to visual areas (Primary_Visual and Dorsal_Stream_Visual) and medial temporal lobe (Medial_Temporal) structurally and functionally.

### Inter-hemispheric SC and RSFC of precuneus

We next analyzed the inter-hemispheric SC and RSFC of precuneus (Supplementary Figs. S3, S4). The inter-hemispheric density map of streamline endpoint and the heatmap of streamline count were shown in Suppl. Fig. S3A, S3B, and S3C, respectively. The regions with significant neural correlates rendered on the contra-lateral hemisphere and the heatmap of RSFC strength were shown in Suppl. Fig. S4A, S4B, and S4C, respectively. To compare the connectional patterns between the inter- and intra-hemisphere, we showed them side by side in Suppl. Fig. S5A–D, respectively. To assess the laterality across hemisphere, we calculated the laterality index (LI) of pairwise connectivity for SC and RSFC in Suppl. Fig. S5E, F, respectively. On the whole, the SC tends to show lower LI values (higher inter-hemispheric connectivity) in the Primary_visual division, while it tends to indicate higher LI values (higher intra-hemispheric connectivity) in the ParaCentral_MidCing, Posterior_Cingulate, and AntCing_MedPFC division (Suppl. Fig. S5E, S5F). In addition, the SC of ventral areas (7m and PCV) tends to represent lower LI values (higher inter-hemispheric connectivity). The RSFC tends to represent higher LI values (higher intra-hemispheric connectivity) in the ParaCentral_MidCing, Superior_Parietal, Inferior_Parietal, and Dorsolateral_Prefrontal division.

### SC-RSFC relationship

**Figure 5:**
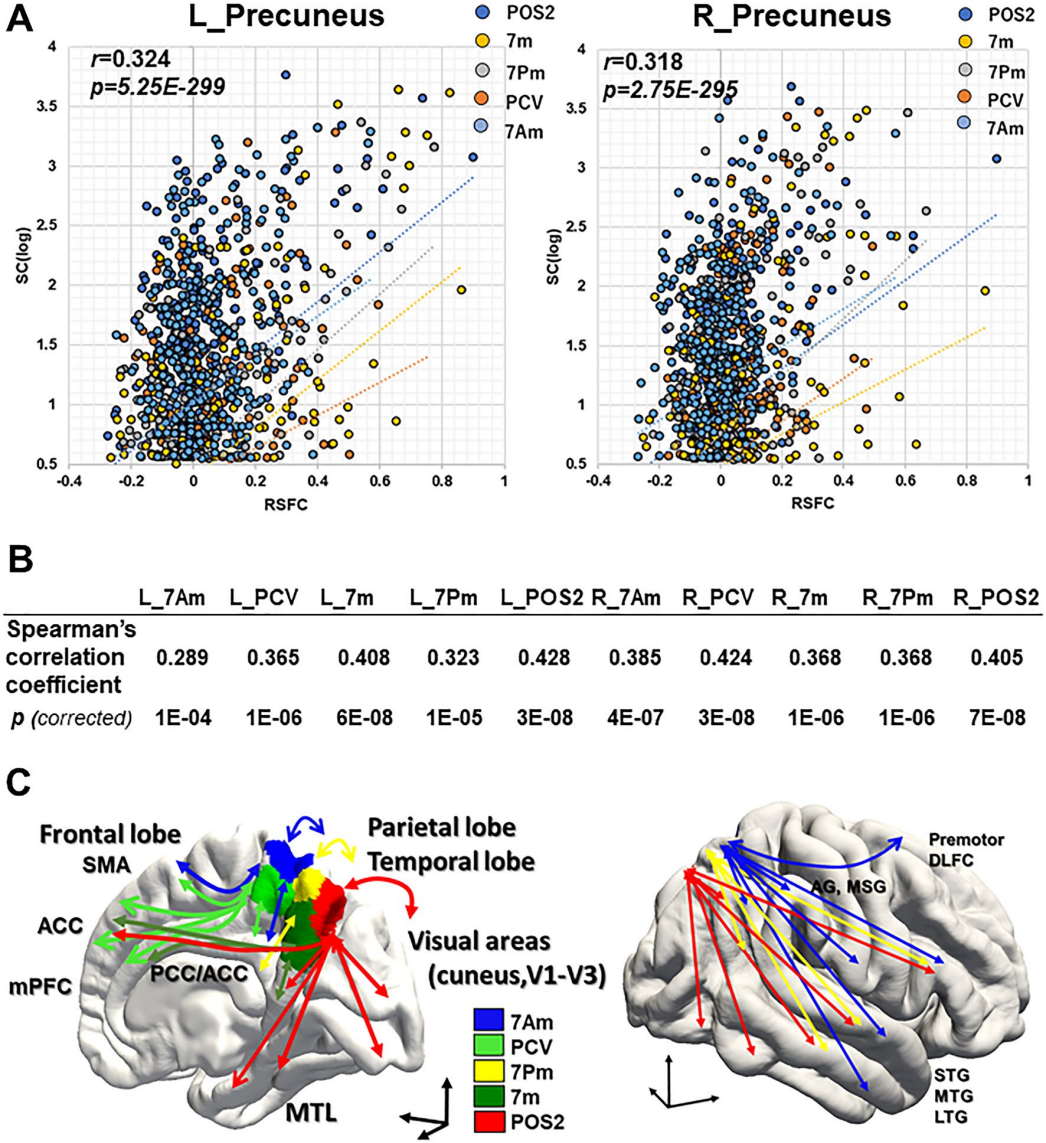
SC-RSFC relationship between precuneus ROIs and cortical areas on HCP MMP atlas. (**A**) Representative subject-level scatter plots (5 × 180 dots /hemisphere) indicating the SC against the RSFC between 5 precuneus ROIs (7Am, PCV, 7m, 7Pm, POS2) and the other cortical areas (180 in HCP MMP atlas) in the left (L) and right (R) hemisphere, respectively (Subject ID: HCP #102614). X-axis, Log10[streamline count/area]; Y-axis, RSFC (Fisher's z transformed pairwise correlation). r; Spearman's rank correlation coefficient between precuneus and whole hemisphere. (**B**) Table showing the group-average Spearman's rank correlation coefficient between each precuneus ROI (7Am, PCV, 7Pm, 7m, POS2) and the other cortical areas (180 in HCP MMP atlas) in the left (L) and right (R) hemisphere, respectively. (**C**) Schematic diagrams showing connectional profile of streamlines by probabilistic tractography, seeded from the 5 precuneus ROIs in the right hemisphere of medial and lateral surface, respectively. ACC, anterior cingulate cortex; AG, angular gyrus; SMG, supra marginal gyrus; SMA, supplementary motor area; mPFC, medial prefrontal cortex; MTL, medial temporal lobe; DLFC, dorsal lateral frontal cortex; STG, superior temporal gyrus; MTG, middle temporal gyrus; LTG, lower temporal lobe.

Then we investigated the relationship between SC and RSFC of precuneus. We first addressed whether there is indeed a relationship between SC and RSFC by calculating the correlation coefficient of the whole-brain at subject level. In Fig. 5A, we showed a representative subject’s SC-RSFC scatter plots by plotting the SC (log streamline count per area) and RSFC (Fisher's z transformed pairwise ROI correlation) in the left (L) and right (R) hemisphere, respectively. Examining all pairwise connections in the whole-brain, we found there is a SC-RSFC relationship (Spearman’s correlation coefficient = 0.282–0.363[mean; 0.322 ± 0.019] at subject level, all *p* < 0.05 corrected) (Suppl. Fig. S6). Next, the SC-RSFC correlation coefficient was calculated between each precuneus ROI and whole-hemisphere at group level (Spearman’s correlation coefficient, L_7Am, 0.289; L_PCV, 0.365; L_7Pm, 0.323; L_7m, 0.408; L_POS2, 0.428; R_7Am, 0.385; R_PCV, 0.424; R_7Pm, 0.368; R_7m, 0.368; R_POS2, 0.405, all *p* < 0.05 corrected) (Fig. 5B, Suppl. Fig. S7). Finally, we here showed schematic diagrams to summarize the structural connection profile by probabilistic tractography, in which the connection patterns of streamlines were described, respectively (Fig. 5C).

## Discussion

In the current study, we showed the characteristic SC of precuneus by probabilistic tractography and the SC-RSFC relationship based on the HCP MMP atlas.

It is generally hypothesized the patterns of FC reflect the underlying anatomical connections^40^. The precuneus is associated with highly integrated cognitive functions, including visuospatial processing, episodic memory retrieval, self-relevant processing, and consciousness^1,2^. Hagmann et al. (2008) identified the precuneus as a structural core of the human cerebral cortex by diffusion spectrum imaging (DSI) and theoretical graph analyses. The precuneus, along with the parietal and posterior medial regions, is highly connected and central, which form a structural core of the human brain^41^. In addition, the precuneus is functionally related to multiple paralimbic networks that comprise subsystems of the default mode network (DMN)^42^. In this context, our results revealed its characteristic connectional profile as a structural core of the human brain.

The precuneus is located in the midline cortical regions, which includes the medial prefrontal cortex (mPFC), posterior cingulate/retrosplenial cortex (PCC/RSC), and para-hippocampus. These regions reportedly play a key role in self-awareness, self-relevant information, and autobiographical memory^2,43–45^. Freton et al.^46^ showed the role of the precuneus in egocentric spatial processing in which the first-person perspective was related to the anterior part of the right precuneus. Wu et al.^47^ showed the anterior precuneus is relevant to the recoverable unconscious states as a cortical hub. Our tractography results indicated the anterior precuneus (7Am) has extensive connections to the frontal, parietal, and temporal lobes (e.g., mPFC, PCC/RSC, and para-hippocampus), which could support the neural networks of self-related processes, memory, and consciousness. The current study revealed the POS2 area is specifically connected to adjacent primary, early, and high-order visual areas. The POS2 also harbors strong connections with the lateral temporal lobe and the medial temporal lobe (MTL) involved in memory processes^15^. Since the POS2 is a possible cognitive control hub for the multi-demand (MD) network that is activated in multiple tasks^48^, we assume the POS2 could be a hub to connect visual areas with multiple cognitive networks.

This study showed the SC-RSFC correlation of the precuneus to some extent, which is consistent with previous report^41^. The precuneus is considered as a node in DMN, within which the nodes have strong SC and RSFC. In contrast, the strong FC often exist between regions with no direct anatomical connection^16^. The ventral precuneus (PCV area) has weak SC but strong FC with the lateral cortical surface (e.g., TPO division) (Figs. 2C, 3C). This might be in part due to the crossing-fiber problem of tractography, which reflects the difficulty of tracking fibers through tight white matter regions with multiple fibers crossing. Especially, TPO (temporo-parietal-occipital) region is one of areas where several major fiber tracts intersect, including the superior longitudinal fasciculus (SLF) and middle longitudinal fasciculus (MLF). Overall, the SC tends to show lower laterality index (LI) values (higher inter-hemispheric connectivity) in the Primary_visual division, while it tends to show higher LI values (higher intra-hemispheric connectivity) in the ParaCentral_MidCing, Posterior_Cingulate, and AntCing_MedPFC division (Suppl. Fig. S5E, S5F). The SC of ventral areas (7m and PCV) tends to represent lower LI values (higher inter-hemispheric connectivity). The RSFC tends to represent higher LI values (higher intra-hemispheric connectivity) in the ParaCentral_MidCing, Superior_Parietal, Inferior_Parietal, and Dorsolateral_Prefrontal division. We assume the ventral areas (7m, PCV) might have more inter-hemispheric connections via commissural fibers (i.e., corpus callosum). Furthermore, we suggest that the precuneus might be highly connected to the adjacent posterior cingulate cortex and medial frontal cortices via the U-fibers and cingulate bundles, respectively, which may lead to higher intra-hemispheric RSFC there. Overall, these results might reflect the homotopic connectivity (HC), which refers to the homotopy between mirror inter-hemispheric areas observed at resting state^49,50^.

Human cerebral cortex forms large-scale networks with complex patterns of divergent and convergent connectivity. In particular, connectivity patterns among association areas harbor the presence of large-scale circuits without clear hierarchical relations, which is made up of multiple interdigitated association networks^42,51^. The precuneus is an association area located on the posteromedial cortex, bordering the default, sensorimotor, dorsal and ventral attention, frontoparietal, and visual network of the principal resting-state networks (RSNs)^51^. The functional networks derived from RSFC represents a good agreement with known functional brain systems^52^. Although it is not straightforward, the structural connection profile could shape and regulate the functional network^41,53,54^. Collectively, the characteristic cortico-cortical connections of the precuneus in the current study could support its integrated cognitive functions.

Using retrograde and anterograde tracer technique, Leichnetz ^55^ reported cortical and subcortical connection profiles of the medial posterior parietal cortex (area 7m) in non-human primates (Cebus and macaque monkeys). It showed the cortico-cortical connections of area 7 m with the adjacent lateral parietal lobe (SPL, IPL), frontal lobe (dorsal prefrontal cortex, premotor area, frontal eye field [FEF], ACC), temporal lobe (e.g., superior temporal sulcus), and adjacent cingulate cortices (PCC/RSC). Our probabilistic tractography results are well consistent with these axon tracing experiments in non-human primates.

Margulies et al.^16^ showed the human precuneus consists of three distinct functional sub-divisions, similar to those of macaque monkeys: anterior, central, and posterior precuneus. The anterior part is a sensorimotor region, while the central part is a cognitive/associative region. The posterior precuneus is connected functionally with adjacent visual cortical regions. Those functional sub-division by rs-fMRI analyses^16^ is well consistent with the SC patterns of our tractography data.

Several issues need to be further addressed. Despite the advancements in methodological techniques and applications, we have no consensus on the best methodology in tractography and fMRI analysis. Our connectome study has utilized the predefined atlas. The parcellation of brain appropriately and precisely is a challenging task at the present time. There are several potential atlases to select from, which affects all subsequent analysis results. The tractography application utilized the grey matter (GM)/white matter (WM) interface as network nodes to build SC, while RSFC analyzed the correlation between distal GM areas as ROIs. This difference might result in the inaccurate estimation of SC-RSFC relation. Further, our tractography approach using dMRI is based on voxel-based analysis. Generally, the trajectory of long-range fiber tracts varies substantially across subjects. The co-registration algorithms inaccurately align fiber tracts due to variation in tract size and shape, which leads to the insufficient precision of voxel-based analysis at the subject level^56,57^. Although no accounting for the geometrical distance, sparsity, and the indirect anatomical connection in the present study, these factors might affect the assessment of SC-RSFC relationship^4^.

In conclusion, our probabilistic tractography results revealed the characteristic cortico-cortical connections of the precuneus that is correlated with the RSFC. The characteristic anatomical connections in the current study could support its integrated cognitive functions.

## Supplementary Information

Supplementary Figures.

## Data Availability

The datasets used and analyzed during the current study available from the corresponding author on reasonable request.

## Abbreviations

MRI: Magnetic resonance imaging
fMRI: Functional MRI
dMRI: Diffusion-weighted MRI
rs-fMRI: Resting-state functional MRI
HCP MMP: Human Connectome Project Multi-modal Parcellation
SC: Structural connectivity
FC: Functional connectivity
RSFC: Resting-state functional connectivity
ROI: Region of interest
RSNs: Resting-state networks
FDR: False Discovery Rate
