# Supplementary material for: Characteristic cortico-cortical connection profile of human precuneus revealed by probabilistic tractography
Source: Sci Rep. 2023 Feb 2;13:1936. doi: 10.1038/s41598-023-29251-2 (PMC9895448; doi:10.1038/s41598-023-29251-2)

Supplementary Fig.S1.

Pipeline for density map of streamline rendered on group-average white surface.

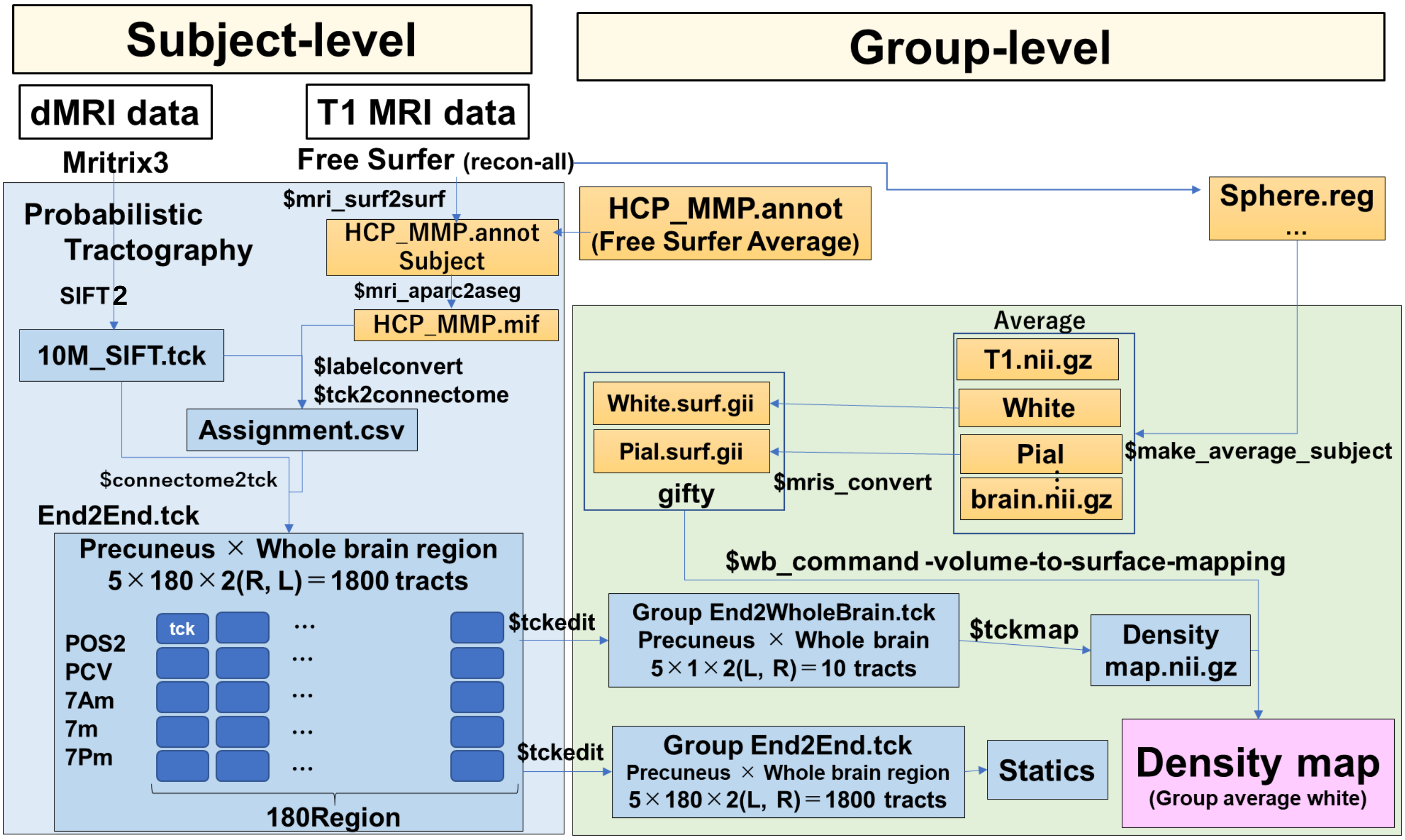

$, command line.

Supplementary Fig.S2.

List of 22 Cortical Divisions for 180 cortical areas in HCP MMP atlas

ID	22 Cortical Divisions	180 Cortical areas (HCP_MMP)															
1	Primary_Visual	V1															
2	Early_Visual	V2	V3	V4													
3	Dorsal_Stream_Visual	V6	V3A	V7	IPS1	V3B	V6A										
4	Ventral_Stream_Visual	V8	FFC	PIT	VMV1	VMV3	VMV2	VVC									
5	MT+_Complex	MST	LO1	LO2	MT	PH	V4t	FST	V3CD	LO3							
6	SomaSens_Motor	4	3b	1	2	3a											
7	ParaCentral_MidCing	5m	5mv	23c	5L	24dd	24dv	SCEF	6ma	6mp							
8	Premotor	FEF	PEF	55b	6d	6v	6r	6a									
9	Posterior_Opercular	43	OP4	OP1	OP2-3	FOP1											
10	Early_Auditory	A1	52	RI	PFcm	PBelt	MBelt	LBelt									
11	Auditory_Association	TA2	STGa	A5	STSda	STSdp	STSvp	A4	STSva								
12	Insula_FrontalOperc	PoI2	FOP4	MI	Pir	AVI	AAIC	FOP3	FOP2	PoI1	Ig	FOP5	PI				
13	Medial_Temporal	EC	PreS	H	PeEc	PHA1	PHA3	TF	PHA2								
14	Lateral_Temporal	TGd	TE1a	TE1p	TE2a	TE2p	PHT	TGv	TE1m								
15	TPO	PSL	STV	TPOJ1	TPOJ2	TPOJ3											
16	Superior_Parietal	7Am	7Pm	7AL	7Pl	7PC	LIPv	VIP	MIP	LIPd	AIP						
17	Inferior_Parietal	PFt	PGp	IP2	IP1	IP0	PFop	PF	PFm	PGi	PGs						
18	Posterior_Cingulate	RSC	PCV	7m	POS1	POS2	23d	v23ab	d23ab	31pv	ProS	DVT	31pd	31a			
19	AntCing_MedPFC	p24pr	33pr	a24pr	p32pr	a24	d32	8BM	p32	10r	9m	10v	25	s32	pOFC	a32pr	p24
20	OrbPolaFrontal	47m	10d	a10p	10pp	11l	13l	OFC	47s	p10p							
21	Inferior_Frontal	44	45	47l	a47r	IFJa	IFJp	IFSp	IFSa	p47r							
22	Dorsolateral_Prefrontal	SFL	8Av	8Ad	8BL	9p	8C	p9-46v	46	a9-46v	9-46d	9a	i6-8	s6-8			

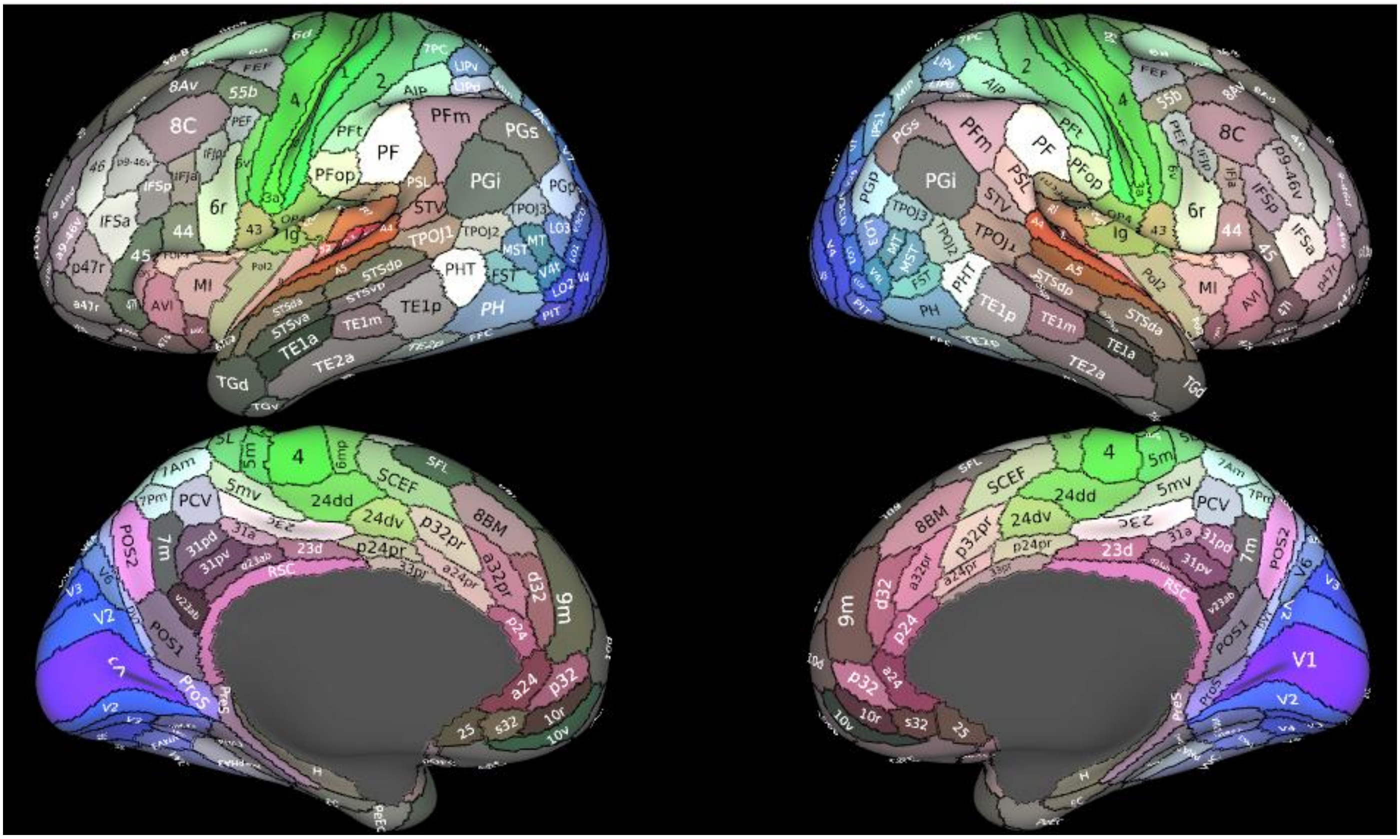

Data from **BALSA (Brain Analysis Library of Spatial maps and Atlases)**

Scene File: HCP_S1200_GroupAvg_BALSA.scene **Reference:** Nature. 536(7615):171-178

Supplementary Fig.S3. Inter-hemispheric Structural Connectivity

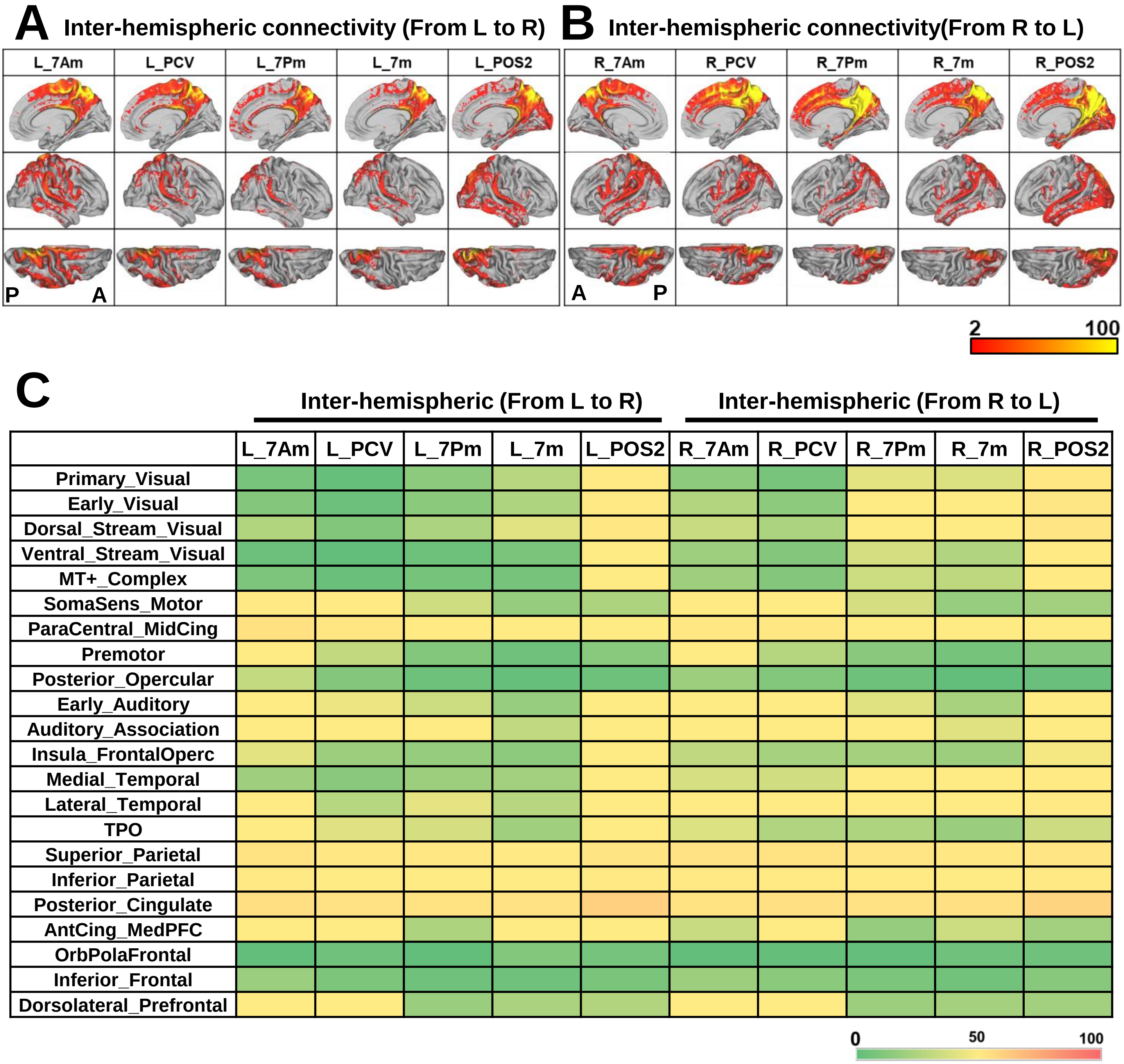

(A) (B) Inter-hemispheric endpoint density map of streamlines, seeded from each precuneus ROI to contra-lateral hemisphere by probabilistic tractography. (A), from left (L) to right (R) hemisphere; (B), from R to L hemisphere. Brain regions representing the density map of streamline endpoint, projected onto the group-average template brain (white matter surface). Medial, lateral, and top view of hemisphere were shown in each lane. The color scale bar represents the group-average endpoint counts/voxel (range; 2-100). A; anterior, P; posterior.

(C) The color map showing the intra-hemispheric connection matrix of streamline count to connect the 5 precuneus ROIs and the 22 Cortical Divisions of HCP MMP atlas. The rows show the 22 Cortical Divisions of HCP MMP atlas, while the lines show the 5 seed regions of precuneus in the left (L) and right (R) hemisphere, respectively. The color scale bar indicates the ratio (%) of maximum streamline count (i.e., R_Posterior_Cingulate - R_POS2)

The details of 22 Cortical Divisions in HCP MMP atlas are shown in Supplementary Fig.S2 with the abbreviations.

Supplementary Fig.S4. Inter-hemispheric Functional Connectivity

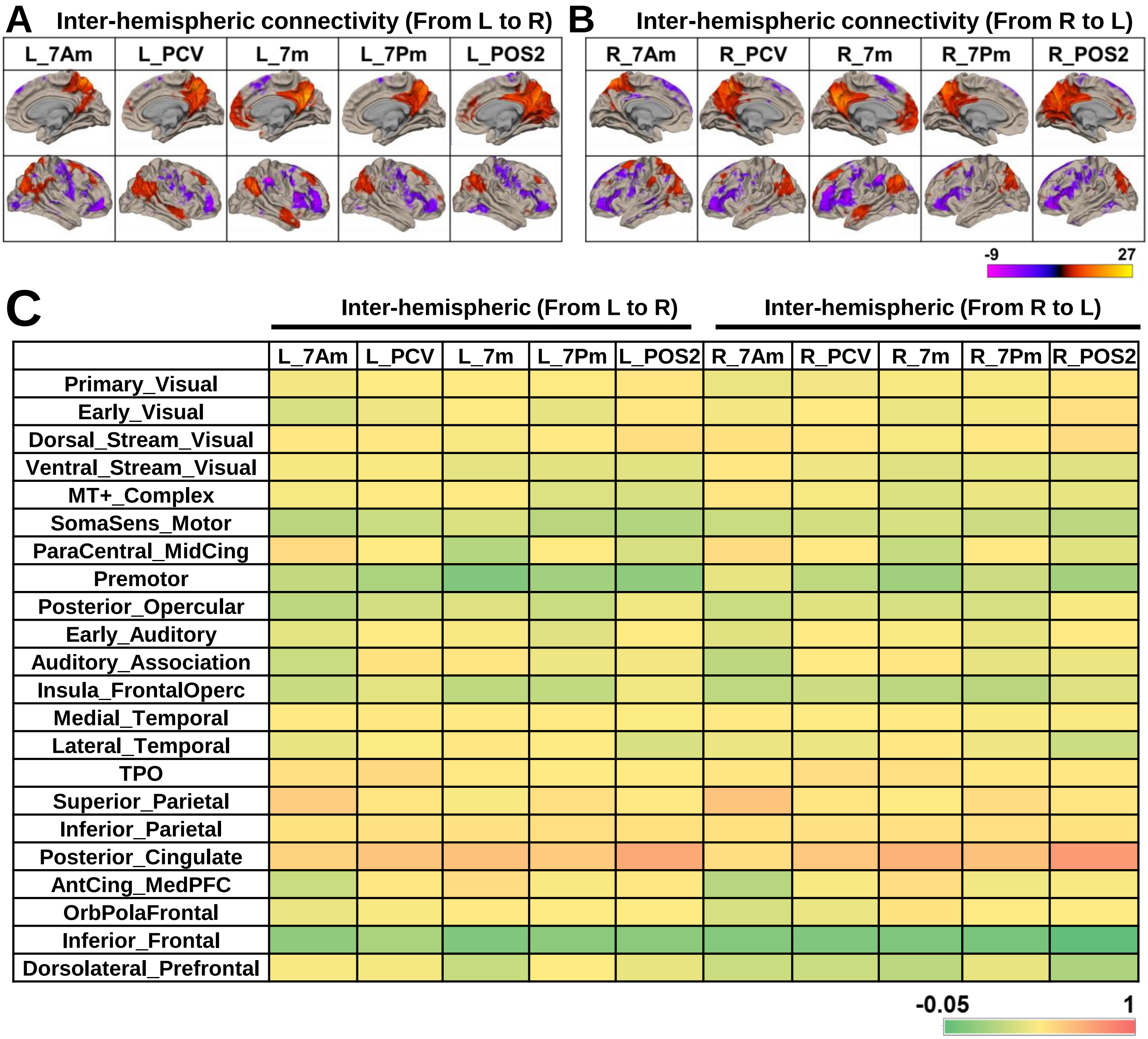

(A) (B) The seed-based resting-state functional connectivity (RSFC) of each precuneus ROI in the left (A) and right (B) hemisphere, respectively. Brain regions representing neural correlates with each precuneus ROI in rs-fMRI analysis (p < 0.001 uncorrected, p <0.05 cluster-level FDR corrected). The regions with significant neural correlates were rendered on the template brain (white matter surface). Color bar represents T-values, in which warmer colors indicate higher T-values.

(C) Group-average connection matrix showing the intra-hemispheric functional connectivity strength between 5 precuneus ROI and 22 Cortical_Divisions of HCP MMP atlas in the left (L) and right (R) hemisphere, respectively. The color scale bar indicates the group-average Fisher-Z transformed correlation. The rows show the 22 Cortical Divisions of HCP MMP atlas, while the columns show the 5 seed regions of precuneus per hemisphere. The details of 22 Cortical Divisions in HCP MMP atlas were shown in Supplementary Fig.S2 with the abbreviations.

Supplementary Fig.S5. Comparison between Inter- and Intra-hemispheric connectivity

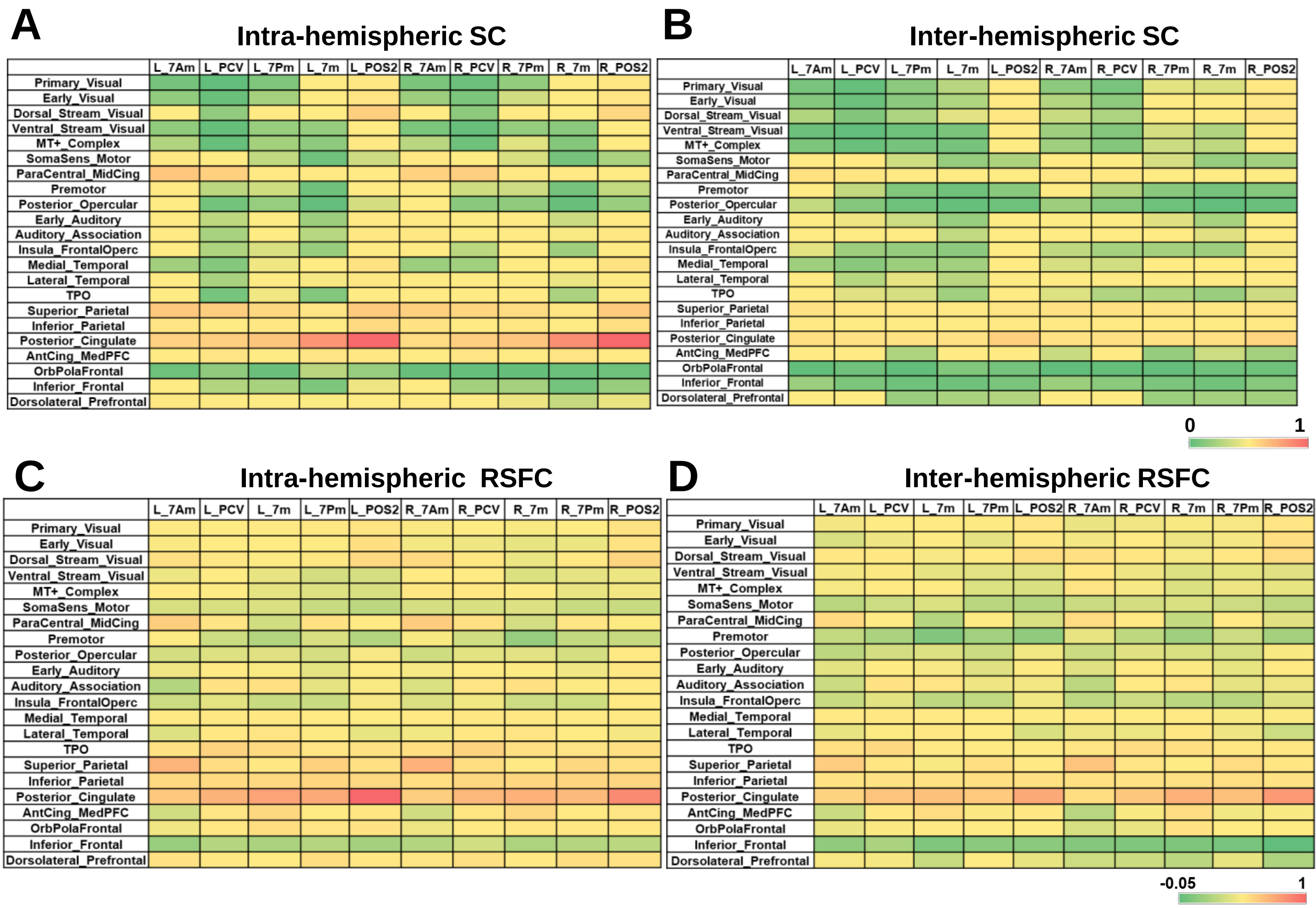

E Laterality Index (LI) of SC

	L_7Am	R_7Am	L_PCV	R_PCV	L_7m	R_7m	L_7Pm	R_7Pm	L_POS2	R_POS2
Primary_Visual	0.001	-0.160	-0.101	-0.210	-0.055	-0.266	0.030	-0.411	-0.053	-0.090
Early_Visual	0.136	-0.114	0.124	-0.229	0.285	-0.089	0.197	-0.319	0.336	-0.068
Dorsal_Stream_Visual	0.487	0.311	-0.006	-0.236	0.249	-0.242	0.552	0.236	0.704	0.460
Ventral_Stream_Visual	0.336	-0.245	0.292	-0.498	0.418	-0.384	0.414	-0.410	0.482	-0.095
MT+_Complex	0.344	0.130	0.056	-0.374	0.186	-0.465	0.305	-0.054	0.515	0.050
SomaSens_Motor	0.097	0.203	-0.365	-0.312	-0.385	-0.351	-0.051	-0.046	0.085	0.010
ParaCentral_MidCing	0.503	0.674	0.592	0.723	0.508	0.500	0.260	0.439	0.281	0.301
Premotor	0.398	0.433	-0.057	0.037	0.009	-0.171	0.346	0.329	0.430	0.310
Posterior_Opercular	0.319	0.441	-0.204	-0.033	-0.089	-0.066	0.360	0.278	0.482	0.375
Early_Auditory	0.278	0.557	-0.294	-0.027	-0.044	-0.030	0.326	0.477	0.516	0.468
Auditory_Association	0.067	0.556	-0.422	0.118	-0.133	-0.039	0.171	0.479	0.404	0.485
Insula_FrontalOperc	0.426	0.539	0.105	0.029	0.102	-0.043	0.257	0.363	0.227	0.392
Medial_Temporal	0.073	-0.301	-0.139	-0.425	0.688	0.481	0.366	-0.098	0.801	0.623
Lateral_Temporal	0.280	0.264	-0.141	-0.183	0.159	-0.306	0.349	0.035	0.424	0.053
TPO	-0.164	0.669	-0.590	0.286	-0.366	0.119	-0.050	0.573	0.236	0.522
Superior_Parietal	0.781	0.693	0.031	-0.195	-0.029	-0.284	0.618	0.436	0.669	0.662
Inferior_Parietal	0.308	0.518	-0.324	-0.095	-0.105	-0.129	0.348	0.446	0.616	0.569
Posterior_Cingulate	-0.036	0.224	0.623	0.748	0.883	0.909	0.303	0.483	0.737	0.796
AntCing_MedPFC	0.308	0.540	0.726	0.853	0.785	0.899	0.638	0.640	0.686	0.692
OrbPolaFrontal	0.377	-0.008	0.418	-0.017	0.448	-0.191	0.460	-0.176	0.385	-0.103
Inferior_Frontal	0.658	0.429	0.382	0.101	0.207	-0.117	0.550	0.189	0.580	0.137
Dorsolateral_Prefrontal	0.529	0.570	0.412	0.328	0.319	0.150	0.450	0.368	0.446	0.254

F Laterality Index (LI) of RSFC

	L_7Am	R_7Am	L_PCV	R_PCV	L_7m	R_7m	L_7Pm	R_7Pm	L_POS2	R_POS2
Primary_Visual	0.254	0.177	0.005	0.218	0.138	0.059	0.230	0.130	0.014	0.138
Early_Visual	0.321	-0.052	0.273	-0.270	-0.014	-0.051	0.216	-0.023	0.308	-0.230
Dorsal_Stream_Visual	0.305	0.247	0.046	-0.023	0.055	-0.100	0.097	0.007	0.166	0.219
Ventral_Stream_Visual	-0.117	-0.103	0.035	0.242	0.029	-0.044	-0.139	-0.012	-0.103	0.161
MT+_Complex	0.157	-0.116	-0.095	0.275	-0.326	0.362	-0.026	-0.026	-0.026	0.080
SomaSens_Motor	0.251	0.107	0.130	0.074	-0.001	0.033	0.156	-0.050	0.026	0.118
ParaCentral_MidCing	0.267	0.380	0.300	0.414	0.150	0.143	0.428	0.235	0.386	0.192
Premotor	0.444	0.149	0.244	0.215	0.302	-0.004	0.390	-0.062	0.210	0.258
Posterior_Opercular	0.195	0.076	0.172	0.029	-0.012	0.072	0.129	-0.011	0.053	0.182
Early_Auditory	0.083	0.207	0.017	-0.030	-0.050	-0.003	0.055	0.061	0.080	0.189
Auditory_Association	-0.199	0.210	-0.031	0.382	0.153	0.110	-0.093	0.186	0.107	0.237
Insula_FrontalOperc	-0.035	0.234	0.001	0.310	0.094	0.272	0.106	0.256	0.121	0.422
Medial_Temporal	0.041	0.067	0.004	0.216	0.057	0.113	0.065	0.223	0.026	0.147
Lateral_Temporal	-0.082	-0.002	0.178	0.318	0.219	0.246	0.025	0.143	0.193	0.374
TPO	-0.120	0.288	0.127	0.270	0.366	-0.124	0.199	0.128	0.323	0.275
Superior_Parietal	0.350	0.206	0.389	0.268	0.242	0.117	0.425	0.100	0.419	0.178
Inferior_Parietal	0.297	0.026	0.226	0.374	0.237	0.284	0.241	0.239	0.249	0.416
Posterior_Cingulate	0.234	0.381	0.192	0.166	0.261	-0.007	0.333	0.058	0.332	0.091
AntCing_MedPFC	0.087	0.222	-0.012	0.418	0.110	0.216	0.040	0.254	0.153	0.415
OrbPolaFrontal	0.051	0.255	0.212	0.302	0.479	-0.087	0.285	0.140	0.390	0.164
Inferior_Frontal	0.055	0.211	0.073	0.519	0.247	0.313	0.279	0.374	0.155	0.442
Dorsolateral_Prefrontal	0.468	0.538	0.161	0.758	0.329	0.550	0.411	0.670	0.341	0.825

-0.6 1

(A) (B) The table shows the structural connectivity of intra- and inter-hemisphere between the precuneus ROIs and the 22 Cortical_Divisions, respectively. For normalization, the values (streamline count between pairwise connection) were converted to Log10 after dividing by the maximum value (i.e., Intra-hemisphere: R_POS2, Inter-hemisphere: L_POS2).
(C)(D) The table shows the resting-state functional connectivity (RSFC, pairwise Z-transformed correlation coefficient) of intra- and inter-hemisphere between the 5 precuneus ROIs/hemisphere and the 22 Cortical_Divisions, respectively.
(E)(F) The table shows the Laterality Index (LI) for the pairwise connectivity of SC and RSFC, respectively.

Supplementary Fig.S6.   SC-RSFC correlation coefficient at subject level

Spearman's rank correlation coefficient (*r*) for SC-RSFC of each subject

	Whole Brain		L_hemisphere(intra)		R_hemisphere(intra)	
**Subject**	***r***	p-value	***r***	p-value	***r***	p-value
**102614**	0.282	0	0.324	5.25E-299	0.318	2.75E-295
**103212**	0.331	0	0.365	0	0.350	0
**103818**	0.345	0	0.408	0	0.366	0
**104012**	0.313	0	0.362	0	0.360	0
**104416**	0.319	0	0.376	0	0.346	0
**104820**	0.332	0	0.368	0	0.381	0
**105014**	0.338	0	0.369	0	0.400	0
**105115**	0.317	0	0.402	0	0.332	0
**106521**	0.294	0	0.347	0	0.353	0
**106824**	0.331	0	0.383	0	0.370	0
**107018**	0.335	0	0.382	0	0.367	0
**107321**	0.296	0	0.382	0	0.353	0
**110411**	0.356	0	0.388	0	0.389	0
**111009**	0.319	0	0.352	0	0.344	0
**111312**	0.363	0	0.400	0	0.406	0
**111413**	0.315	0	0.346	0	0.369	0
**111514**	0.315	0	0.336	0	0.347	0
**111716**	0.291	0	0.315	3.53e-316	0.352	0
**112314**	0.336	0	0.393	0	0.322	0
**112920**	0.356	0	0.388	0	0.363	0
**113316**	0.333	0	0.359	0	0.368	0
**113619**	0.316	0	0.357	0	0.345	0
**114621**	0.312	0	0.350	0	0.346	0
**116726**	0.324	0	0.363	0	0.351	0
**117324**	0.326	0	0.374	0	0.364	0
**117930**	0.329	0	0.014	0.129	0.352	0
**118124**	0.299	0	0.354	0	0.323	4.86E-273
**118225**	0.327	0	0.361	0	0.385	0
**118528**	0.309	0	0.360	0	0.338	1.976263e-323
**118730**	0.326	0	0.368	0	0.367	0
**119126**	0.296	0	0.349	0	0.324	0
**119732**	0.319	0	0.356	0	0.327	0
**119833**	0.310	0	0.359	0	0.362	0
**average**	**0.322**		**0.355**		**0.356**	

Supplementary Fig.S7. SC-RSFC correlation coefficient of each precuneus ROI at group level

	L_7Am	L_PCV	L_7m	L_7Pm	L_POS2	R_7Am	R_PCV	R_7m	R_7Pm	R_POS2
**Spearman's correlation coefficient**	**0.289**	**0.365**	**0.408**	**0.323**	**0.428**	**0.385**	**0.424**	**0.368**	**0.368**	**0.405**
p (uncorrected)	**1E-04**	**8E-07**	**2E-08**	**1E-05**	**3E-09**	**2E-07**	**7E-09**	**7E-07**	**7E-07**	**3E-08**

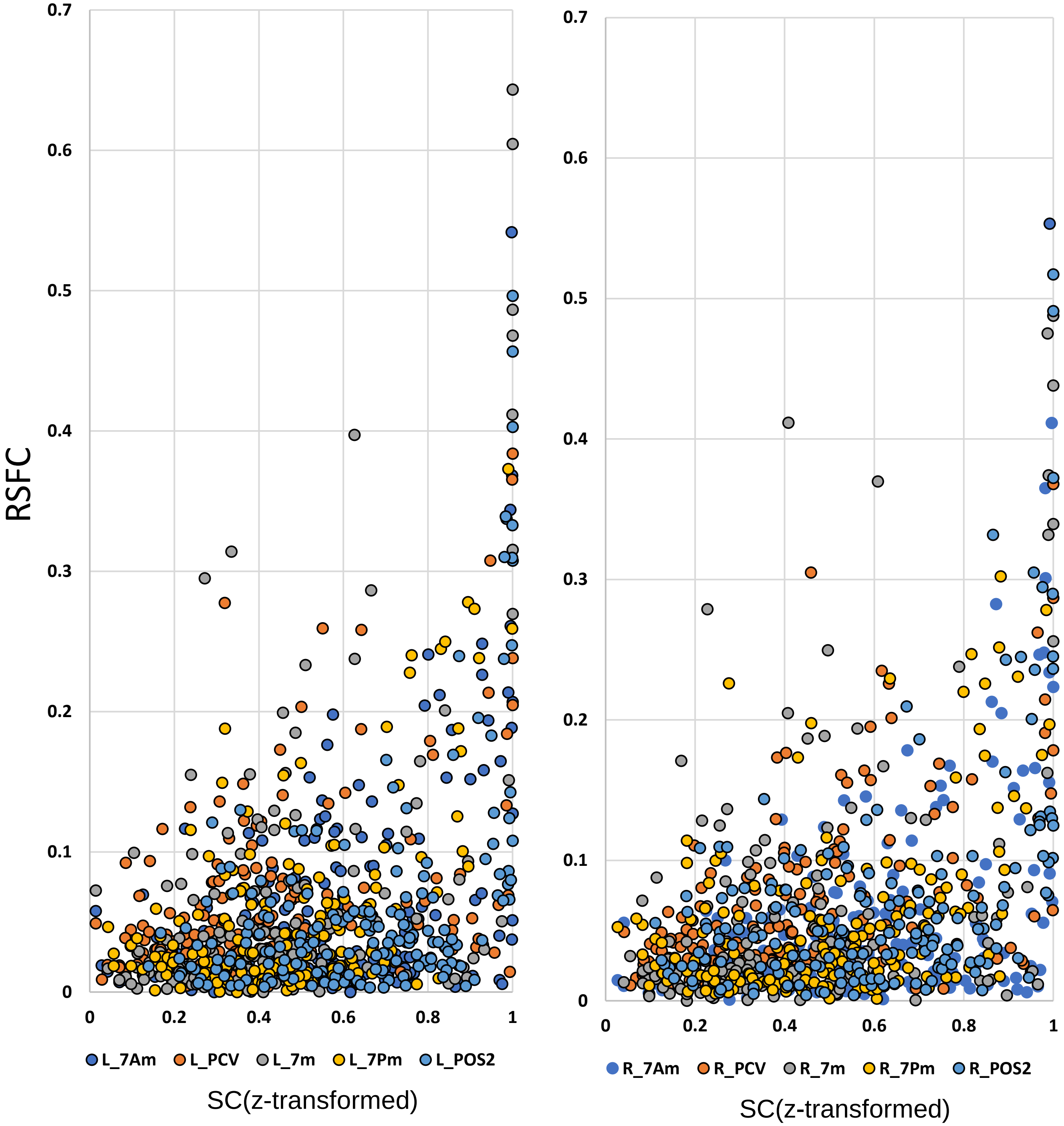

Supplement: Supplementary file 1 — Supplementary Figures. [file 41598_2023_29251_MOESM1_ESM.pdf]
